# YC-1 enhances the anti-tumor activity of sorafenib through inhibition of signal transducer and activator of transcription 3 (STAT3) in hepatocellular carcinoma

**DOI:** 10.1186/1476-4598-13-7

**Published:** 2014-01-13

**Authors:** Jian Kong, Fandong Kong, Jun Gao, Qiangbo Zhang, Shuying Dong, Fang Gu, Shan Ke, Bing Pan, Qiang Shen, Huichuan Sun, Lemin Zheng, Wenbing Sun

**Affiliations:** 1 Department of Hepatobiliary Surgery, Beijing Chaoyang Hospital, Capital Medical University, Beijing 100043, People’s Republic of China; 2 The Institute of Cardiovascular Sciences and Institute of Systems Biomedicine, School of Basic Medical Sciences, Peking University Health Science Center, Key Laboratory of Molecular Cardiovascular Sciences of Education Ministry, and Key Laboratory of Cardiovascular Molecular Biology and Regulatory Peptides of Health Ministry, Beijing 100191, People’s Republic of China; 3 Liver Cancer Institute, Zhongshan Hospital, Fudan University, Shanghai 200032, People’s Republic of China; 4 Department of General Surgery, Qilu Hospital, Shandong University, Jinan 250012, People’s Republic of China; 5 Department of Obstetrics and Gynecology, Chinese PLA General Hospital, Beijing 100853, People’s Republic of China

**Keywords:** YC-1, Sorafenib, Hepatocellular carcinoma, STAT3

## Abstract

**Background:**

Traditional systemic chemotherapy does not provide survival benefits in patients with hepatocellular carcinoma (HCC). Molecular targeted therapy shows promise for HCC treatment, however, the duration of effectiveness for targeted therapies is finite and combination therapies offer the potential for improved effectiveness.

**Methods:**

Sorafenib, a multikinase inhibitor, and YC-1, a soluble guanylyl cyclase (sGC) activator, were tested in HCC by proliferation assay, cell cycle analysis and western blot *in vitro* and orthotopic and ectopic HCC models *in vivo*.

**Results:**

*In vitro*, combination of sorafenib and YC-1 synergistically inhibited proliferation and colony formation of HepG2, BEL-7402 and HCCLM3 cells. The combination also induced S cell cycle arrest and apoptosis, as observed by activated PARP and caspase 8. Sorafenib and YC-1 respectively suppressed the expression of phosphorylated STAT3 (p-STAT3) (Y705) in a dose- and time-dependent manner. Combination of sorafenib and YC-1 significantly inhibited the expression of p-STAT3 (Y705) (S727), p-ERK1/2, cyclin D1 and survivin and SHP-1 activity compared with sorafenib or YC-1 used alone in all tested HCC cell lines. *In vivo*, sorafenib-YC-1 combination significantly suppressed the growth of HepG2 tumor xenografts with decreased cell proliferation and increased apoptosis observed by PCNA and PARP. Similar results were also confirmed in a HCCLM3 orthotopic model. There was a reduction in CD31-positive blood vessels and reduced VEGF expression, which suggested a combinational effect of sorafenib and YC-1 on angiogenesis. The reduced expression of p-STAT3, cyclin D1 and survivin was also observed with the combination of sorafenib and YC-1.

**Conclusions:**

Our data show that sorafenib-YC-1 combination is a novel potent therapeutic agent that can target the STAT3 signaling pathway to inhibit HCC tumor growth.

## Background

Hepatocellular carcinoma (HCC) is the sixth most common neoplasm and the third most frequent cause of cancer death [1]. Surgery, local treatment and liver transplantation may provide curative potential for patients with HCC, however, only 10-20% of patients are eligible for curative therapies [2]. Furthermore, traditional systemic chemotherapy does not provide survival benefits in patients with HCC. Molecular targeted therapy shows promise for HCC treatment. Sorafenib (Nexavar^®^) is the first and only molecular targeted therapy approved for use in HCC by the U.S. Food and Drug Administration in 2007. Sorafenib, a multiple kinase inhibitor, displays a remarkable inhibition of Raf-1 and others tyrosine kinases such as vascular endothelial growth factor receptor 2 (VEGFR2), VEGFR3, Flt-3, platelet derived growth factor (PDGF), and fibroblast growth factor recptor-1 (FGFR-1) [3]. Although sorafenib showed survival benefits in large randomized phase III studies, the response rate is actually quite low [4,5]. Sorafenib also causes multiple human toxicities, including use-limiting anorexia, GI bleeds and hand-foot syndrome [5]. Modulation of its actions that result in lessening of the toxicities is a desirable goal. Consequently, the duration of effectiveness for targeted therapies is finite; however, combination therapies offer the potential for improved effectiveness [6].

YC-1 [3-(5′-hydroxymethyl-2′-furyl)-1-benzylindazole] is an effective anti-platelet agent that increases cGMP levels through NO-independent activation of soluble guanylyl cyclase (sGC) and originally developed as a drug in the treatment of platelet-rich thrombosis, vasospasm or male erectile dysfunction [7]. Currently, YC-1 is also used as an inhibitor of hypoxia-inducible factor 1α (HIF-1α) to suppress the tumor growth [8]. YC-1 exhibits anti-proliferative effects against various cancer cell lines including breast cancer [9], epithelial ovarian carcinoma [10], bladder transitional carcinoma [8], prostate cancer [11], oesophageal squamous carcinoma [12], renal carcinoma [13], lung cancer [14], leukemia [15], pancreatic cancer [16], and HCC [17-19]. YC-1 also exhibits potential prospective in treatment for HCC. YC-1 induced cell cycle arrest and apoptosis by activating checkpoint kinases in HCC cells [18,19]. YC-1 also enhanced chemo-sensitivity and eliminated the arsenic trioxide resistance in HCC [20]. Whether the combined use of YC-1 and sorafenib has clinical therapeutic potential for HCC patients has not been determined.

The signal transducer and activator of transcription 3 (STAT3) has been implicated in signal transduction by different cytokines, growth factors and oncogenes. Activated STAT3 plays an important role in tumorigenesis through the upregulation of genes involved in anti-apoptosis, proliferation and angiogenesis [21]. Consequently, STAT3 has emerged as a promising target for cancer therapy, which is pharmacologically safe and effective, and blocking STAT3 activation has the potential for treatment of HCC. Recent findings showed that sorafenib inhibited tumor growth through Raf-MEK-MAPK-independent pathways and STAT3 was a major kinase-independent target of sorafenib in HCC [22], and sorafenib inhibited growth and metastasis of HCC by blocking STAT3 [23]. However, endothelial growth factor receptor (EGFR), which could activate STAT3, has been implicated both in the inherent and acquired resistance to sorafenib [24,25]. A majority of HCC patients do not respond to sorafenib, and most if not all patients who initially respond to sorafenib, subsequently become refractory and develop tumor progression after a few months of sorafenib therapy [26]. Therefore, an approach that improves therapeutic efficacy is urgently needed. Several preclinical studies have revealed the apoptosis-enhancing effects of sorafenib on both hematological and solid tumor cells. YC-1 was also reported to inhibit the activity of STAT3 in HCC [17]. Whether YC-1 could be used to enhance the inhibition of activation of STAT3 of sorafenib needs to be elucidated.

In this article, we asked whether the anti-proliferative property of sorafenib and YC-1 in HCC tumors was synergic when administered in combination, and whether their concomitant use played a role through the inhibition of the STAT3 signaling pathway. We assessed the combined effects of sorafenib and YC-1 on HCC cells and xenograft models. Our results provide a rationale for combined use of sorafenib and YC-1 in HCC.

## Methods

### Materials

Sorafenib was kindly provided by Bayer Pharmaceuticals. YC-1 was obtained from Sigma-Aldrich (Saint Quentin Fallavier, France). Anti-PARP, anti-caspase 8, anti-β-actin, and anti-caspase 9 were obtained from Beyotime (Jiangsu, China). Horseradish peroxidase (HRP)-labeled anti-mouse and anti-rabbit secondary antibodies were from Santa Cruz (Dallas, TX, USA). All other antibodies were purchased from Abcam (Cambridge, TX, USA).

### Cell lines and cell culture

Established human HCC cell lines, HepG2 and BEL-7402 were from the American Type Culture Collection (ATCC; Manassas, VA, USA). HCCLM3 was kindly provided by the Liver Cancer Institute and Zhongshan Hospital (Shanghai, China) [27]. All HCC cells were maintained in high-glucose Dulbecco’s modified Eagle medium (DMEM) supplement with 10% fetal bovine serum (FBS), 100 U/ml penicillin and 100 μg/ml streptomycin (Life Technologies, Cergy Pontoise, France) in a humidified atmosphere of 5% CO_2_ at 37°C. The human normal liver cell line L02 was provided by Cancer Institute & Hospital of Chinese Academy of Medical Sciences, which was cultured in PRMI 1640 supplement with 10% FBS, 100 U/ml penicillin and 100 μg/ml streptomycin. All cell lines were immediately expanded and frozen down such that all cell lines could be restarted every 3 months from a frozen vial of the same batch of cells. No further authentication was done. All cell lines were routinely tested to rule out mycoplasma infection.

### Proliferation assay

Cell proliferation assay was measured using a Cell Counting Kit-8 (CCK-8; Dojindo Laboratories, Shanghai, China) according to the manufacturer’s instructions. Briefly, cells were cultured in 96-well plates at a concentration of 3 × 10^3^/well, incubated for 24 h, and treated with sorafenib and/or YC-1. After 72 h treatment, CCK-8 reagent was added to each well. The absorbance was measured at 450 nm after 2.5 h incubation at 37°C using an automated ELISA plate reader. Any synergistic effects resulting from combination of the compounds were measured using the methods described by Chou and Talalay by using Microsoft Excel software to determine the combination index values (CI > 1: antagonistic effect, CI = 1: additive effect, and CT < 1: synergistic effect) [28].

### Colony formation assay

Briefly, 6-well dishes were seeded with 1 × 10^3^ viable cells and allowed to grow for 24 h. The cells were then incubated in the presence or absence of sorafenib, YC-1 and their combinations for 24 h in complete medium, washed with media, and allowed to grow in complete medium for 2 weeks. The colonies obtained were washed with PBS and fixed in 4% paraformaldehyde for 20 min at room temperature and then washed with PBS followed by staining with crystal violet. The colonies were counted and compared with untreated cells. Three different independent experiments were performed.

### Cell cycle analysis

HCC cells were plated in 6-well plates at 2 × 10^5^ cells/well. Following the designated treatments, cells were harvested by trypsinization and washed with PBS and fixed in ice-cold 75% ethanol overnight at -20°C. Fixed cells were washed, and dissolved in RNAse and subsequently incubated at 37°C for 30 min. Next, cells were stained with propidium iodide (PI) for 30 min. The DNA content of the cells (1 × 10^4^ cells per experimental group) was determined using a BD Accuri C6 flow cytometer (BD biosciences).

### Apoptosis assay

HCC cells (HepG2, BEL-7402 or HCCLM3) were seeded in 6-well culture plates in culture medium with 2% FBS at the concentration of 2 × 10^5^ cells/well. The following day, the cells were treated with sorafenib and/or YC-1 for a 48 h period. After treatment, cells were washed with PBS and fixed with 4% paraformaldehyde followed by staining with Hoechst 33258, and the apoptosis cells were examined by immunofluorescence microscope, or all cells including both floating and attached cell were collected, and the apoptotic cells were detected by Annexin V-FITC Apoptosis Detection Kit (KeyGEN Biotech, Nanjing, China). The cells were stained with Annexin V-FITC and PI according to the supplier’s instructions. Viable and dead cells were detected by a BD Accuri C6 flow cytometer (BD biosciences).

### Gene knockdown using small interfering RNA

SMARTpool small interfering RNAs (siRNA), including control and STAT3 were synthesized by Shanghai GenePharma Co. (Shanghai, China). The procedure has been described previously [29].

### Western blot analysis

Cell lysate protein content was determined using a Bicinchoninic acid (BCA) protein assay kit. Equivalent amounts of whole cell extracts, which were based on total protein content, were subjected to SDS-PAGE gel and transferred to nitrocellulose membranes. The membranes were blocked with 5% non-fat milk for 2 h and then incubated with respective primary antibody overnight at 4°C followed by the incubation with the appropriate HRP-conjugated secondary antibody for 1.5 h at room temperature. Immunoreactivity was detected with SuperSignal West Pico substrate (Thermo scientific, Rockford, IL, USA).

### SHP-1 phosphatase activity

The Rediplate 96 EnzChek^®^Tyrosine Phosphatase Assay Kit (R-22067) was used for SHP-1 activity assay (Molecular Probes, Carlsbad, CA). The SHP-1 phosphatase activity was measured as described before [30].

### Animals

Male BALB/c nu/nu mice (4–6 weeks of age) were obtained from Vital River Laboratories (Beijing, China) and housed under defined flora conditions in individually ventilated sterile microisolator cages. All experimental procedures using these mice were carried out in accordance with protocols approved by the Animal Care and Use Committee of Capital Medical University (Beijing, China).

### Orthotopic and ectopic HCC models

In the orthotopic model, HCCLM3 cells (5 × 10^6^) were suspended in 200 μL serum-free DMEM and matrigel (1:1) and then injected subcutaneously into the upper right flank region of nude mice. When the subcutaneous tumor reached approximately 1 cm in length (approximately 4 weeks after injection), it was removed, minced into small pieces of equal volume (2 × 2 × 2 mm^3^), and transplanted into the livers of 20 nude mice. In the ectopic HCC model, HepG2 cells (5 × 10^6^) were suspended in 200 μL serum-free DMEM and matrigel (1:1) and then injected subcutaneously into the upper right flank region of 20 nude mice. When the tumor reached a mean size of about 100 mm^3^, mice were randomized into each experimental group according to tumor size, to start the treatment with a similar mean size in each group. Mice were treated with sorafenib by oral route (30 mg/kg/day), or intraperitoneal YC-1 (10 mg/kg/day), or combination of sorafenib and YC-1, or DMSO and polyoxyethylenated castor oil as control every day for up to the 24th day. Tumor size was measured with a caliper rule every 3 days. The tumor volume was calculated as follows: TV (mm^3^) = (L × W^2^)/2, where L was the longest and W the shortest radius of the tumor in millimeters. At the end of the experiments, mice were euthanized, blood samples were collected via cardiac puncture, and tumor tissues were removed for fixation in the 4% paraformaldehyde for histologic examination and immunohistochemical staining.

### Immunocytochemistry

Tissues were fixed in 4% paraformaldehyde and subsequently embedded in paraffin. Paraffin-embedded tissue sections were cut into standard 6 μm sections, deparaffinized in xylene and rehydrated through graded alcohol solutions. Antigen retrieval was performed 10 min at 92°C in EDTA (10 mmol/l, pH 8.0) in a water bath. Endogenous peroxidases were inactivated by immersing the sections in 0.3% hydrogen peroxide for 12 min. The sections were blocked with 5% goat serum for 60 min at 37°C. The slides were incubated with primary antibodies for overnight at 4°C. Next, the slides were treated with appropriate HRP-conjugated secondary antibody for 40 min at 37°C and then developed with 3,3′-diaminobenzidine. Finally, the slides were counterstained with hematoxylin and mounted. The slides were examined with Nikon Eclipse Ti microscope under a 200× objective.

### Statistical analysis

All values are expressed as the mean ± SEM. The data were analyzed using Student’s *t* test or the ANOVA test. A P value of <0.05 was considered statistically significant. GraphPad Prism (GraphPad Software Inc., San Diego, California, USA) was used for these analyses.

## Results

### Combination of sorafenib and YC-1 inhibited HCC cell proliferation

**Figure 1:**
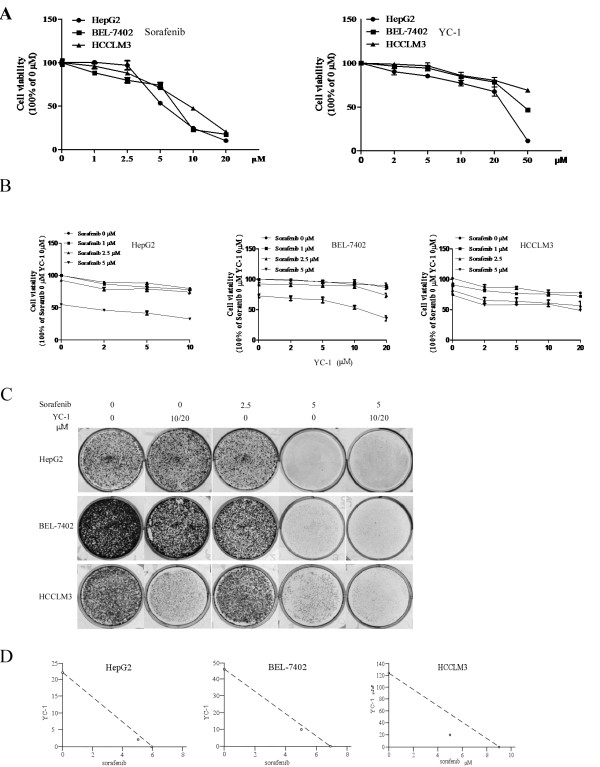
**Sorafenib-YC-1 combination synergistically inhibited proliferation of HCC cells. A**, HepG2, BEL-7402 and HCCLM3 cells were incubated with increasing doses of sorafenib (0–20 μmol/L) or YC-1 (0–50 μmol/L) for up to 72 h. Data were presented as percentages of cell proliferation as determined by CCK-8 assays. **B**, HepG2, BEL-7402 and HCCLM3 cells were incubated with sorafenib (0–5 μmol/L) and/or YC-1 (0–20 μmol/L) for up to 72 h. Data were presented as percentages of cell proliferation as determined by CCK-8 assays. **C**, HepG2, BEL-7402 and HCCLM3 cells were treated sorafenib (2.5 μmol/L or 5 μmol/L) or YC-1 (10 μmol/L or 20 μmol/L) or in combination. The colonies were assessed. **D**, Synergisms of proliferation inhibition of different cell lines were analyzed by isobologram analysis. Error bars represent the SEM of data obtained in at least three independent experiments.

To determine the growth inhibition effect of combination of sorafenib and YC-1, HCC cell lines HepG2, BEL-7402 and HCCLM3 were incubated for 72 h with sorafenib and/or YC-1. Chemical structures of sorafenib and YC-1 were shown in Additional file 1: Figure S1. Sorafenib or YC-1 alone inhibited HCC cell proliferation in a dose-dependent manner (Figure 1A). Moreover, combination of sorafenib and YC-1 significantly suppressed proliferation of HCC cells in a dose-dependent manner (Figure 1B). To determine the long-term combination effect of sorafenib-YC-1 treatment, cells were incubated with sorafenib and/or YC-1 for 24 h, washed with media, and allowed to grow in complete medium for 2 weeks. There was lower number of colonies in the combination compared with other treatments (Figure 1C). In addition, at the ED_50_ doses for both sorafenib and YC-1, we found that CI = 0.93 in HepG2, 0.95 in BEL-7402 and 0.72 in HCCLM3 respectively, suggesting that sorafenib and YC-1 synergistically inhibited proliferation of HCC cells (Figure 1D). These data suggested that combination of sorafenib and YC-1 treatment synergistically suppresses proliferation of HCC cells *in vitro*. Otherwise, we determined the effect of the combination on proliferation of the human normal liver cell line L02. Sorafenib (5 μM) significantly inhibited (66.9% decrease) the proliferation of L02 cells (Additional file 2: Figure S2). Although YC-1 (20 μM) significantly suppressed the proliferation of L02 cells, only 12.1% decrease was observed (Additional file 2: Figure S2). YC-1 could not increase the toxicity of sorafenib to L02 cells (Additional file 2: Figure S2).

### The sorafenib-YC-1 combination induced cell cycle arrest and apoptosis

**Figure 2:**
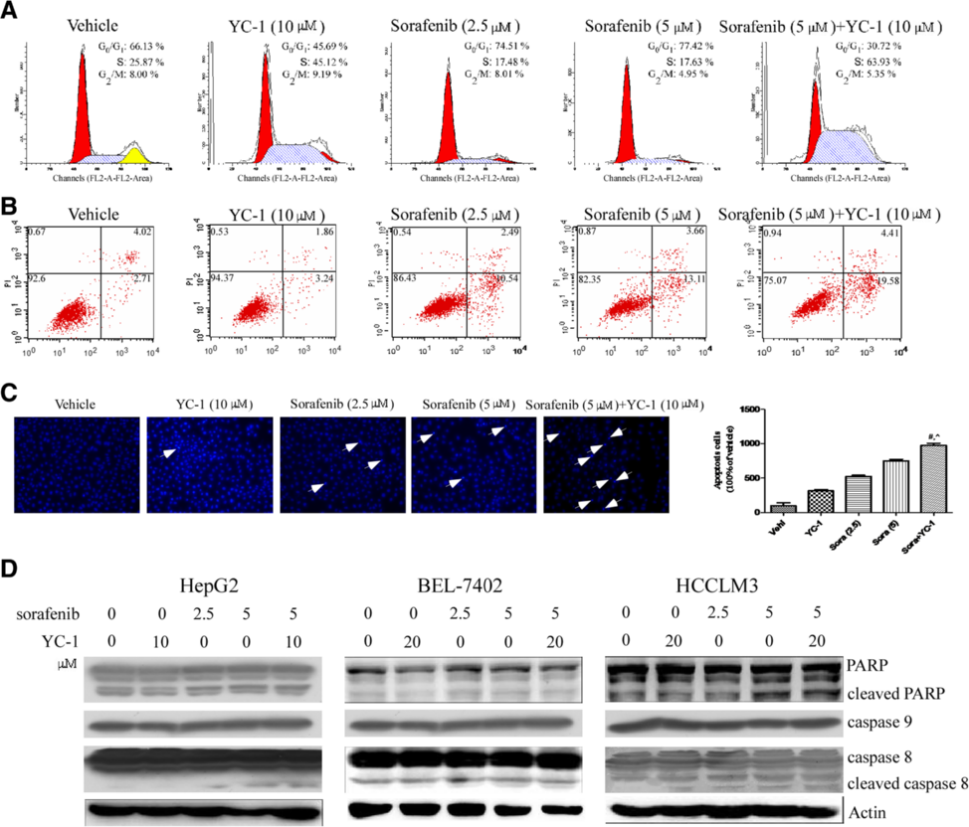
**Combination of sorafenib and YC-1 induced S cell cycle arrest and apoptosis of HCC cells.** HepG2 cells were treated with combination of sorafenib (5 μmol/L) and YC-1 (10 μmol/L) or either drug for 48 h. **A**, Cell cycle was assayed by PI/RNAse staining. **B**, The treated cells were assayed for apoptosis by annexin V/PI staining. **C**, Apoptosis in situ was assessed by Hoechst 33258 staining. **D**, Cell extracts were subjected to western blot analysis and expression of PARP, cleaved PARP, capase 9, capsase 8 and cleaved caspase 8 was detected. Actin served as loading control.

Given their effects on cell proliferation, we then carried out cell-cycle and apoptosis analysis to further characterize sorafenib-YC-1 combination effects. HepG2 cell populations in the G0-G1 and S phases were 30.72% and 63.93% in the combination of sorafenib and YC-1 group, while 77.42% and 17.63% in sorafenib (5 μM) group, and 45.69% and 45.12% in YC-1 group (Figure 2A). These results suggested that sorafenib-YC-1 combination significantly induced S phase arrest compared with sorafenib or YC-1 used alone in HepG2 cells. At 48 h, sorafenib-YC-1 combination significantly induced apoptosis in HepG2 cells (Figure 2B). Hoechst 33258 staining further confirmed results above (Figure 2C). Similar results were also found in BEL-7402 and HCCLM3 cells (data of apoptosis assay not shown) (Additional file 3: Figure S3). Furthermore, we determined the mechanism of cell death. Increased activation of PARP was observed within 24 h in all HCC cells treated with sorafenib-YC-1 combination (Figure 2D). The apoptotic signaling pathways are generally divided into two types: the extrinsic or death receptor pathway, which activates caspase 8, and the intrinsic or mitochondrial pathway, which triggers the activation of caspase 9. Increased activation of caspase 8 not caspase 9 was observed in HCC cells treated with sorafenib-YC-1 combination (Figure 2D). These data suggested that combination of sorafenib and YC-1 is a potent inducer of apoptosis of HCC cells involved in activation of the extrinsic apoptosis pathway.

### The sorafenib-YC-1 combination suppressed STAT3 phosphorylation

**Figure 3:**
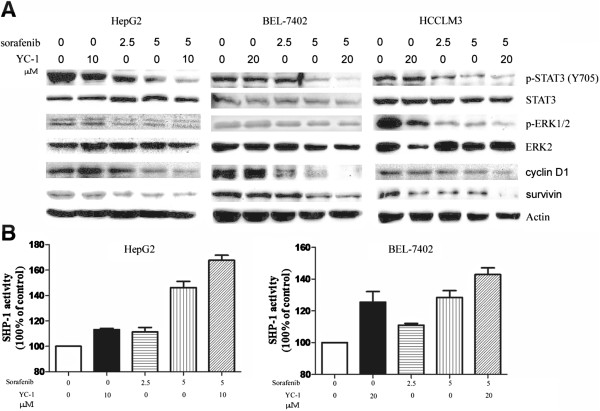
**Combination of sorafenib and YC-1 suppressed the activity of STAT3 and expression of its associated protein.** HepG2, BEL-7402 and HCCLM3 cells were incubated with sorafenib (5 μmol/L) in combination with YC-1 (10 μmol/L or 20 μmol/L) or either drug alone for 24 h. **A**. Cell extracts were subjected to western blot analysis and expression of p-STAT3 (Y705), STAT3, cyclin D1 and survivin was assessed. Actin served as loading control. **B**. SHP-1 activity was measured in HepG2 and BEL-7402 cells.

We investigated the levels of phosphorylated STAT3 and p44/42 MAPK (ERK1/2) in HepG2, BEL-7402 and HCCLM3 cells after sorafenib and/or YC-1 treatment. Phosphorylated STAT3 (p-STAT3) (Y705) and phosphorylated ERK1/2 (p-ERK1/2) were reduced at both an early time point (4 h) and a late time point (24 h) following sorafenib treatment in a dose-dependent manner (Additional file 4: Figure S4). Furthermore, p-STAT3 at Y705 was also decreased at 4 h and 24 h following YC-1 treatment in a dose-dependent manner (Additional file 4: Figure S4). However, p-ERK1/2 was not significantly changed at early time point following YC-1 treatment. And at a late time point, p-ERK1/2 was not changed in HepG2 cells and significantly changed in BEL-7402 only at the concentration of 50 μM YC-1 (Additional file 4: Figure S4). Moreover, p-ERK1/2 was reduced in a dose-dependent manner at a late time point following YC-1 treatment in HCCLM3 cells (Additional file 4: Figure S4). Interestingly, combination of sorafenib and YC-1 significantly inhibited the p-STAT3 (Y705) (S727) and p-ERK1/2 compared with sorafenib or YC-1 used alone at 24 h (Figure 3A and Additional file 5: Figure S5). Sorafenib-YC-1 combination also suppressed the expression of cyclin D1 and survivin in all tested HCC cell lines (Figure 3A).

Furthermore, both YC-1 and sorafenib increased SHP-1 activity in comparison with control cells, and the combination of sorafenib and YC-1 significantly increased SHP-1 activity compared with sorafenib or YC-1 alone in HepG2 and BEL-7402 cells (Figure 3B).

Furthermore, STAT3 siRNA was used to delete STAT3, and the expression of p-STAT3 (Y705) and STAT3 was significantly decreased in HepG2 and BEL-7402 cells (Additional file 6: Figure S6A). Deletion of STAT3 could not rescue HepG2 and BEL-7402 cells from apoptosis. Inversely, silencing STAT3 improved the sensitivity of HepG2 and BEL-7402 cells to the combination of sorafenib and YC-1 (Additional file 6: Figure S6B).

### The sorafenib-YC-1 combination inhibited tumor growth *in vivo*

**Figure 4:**
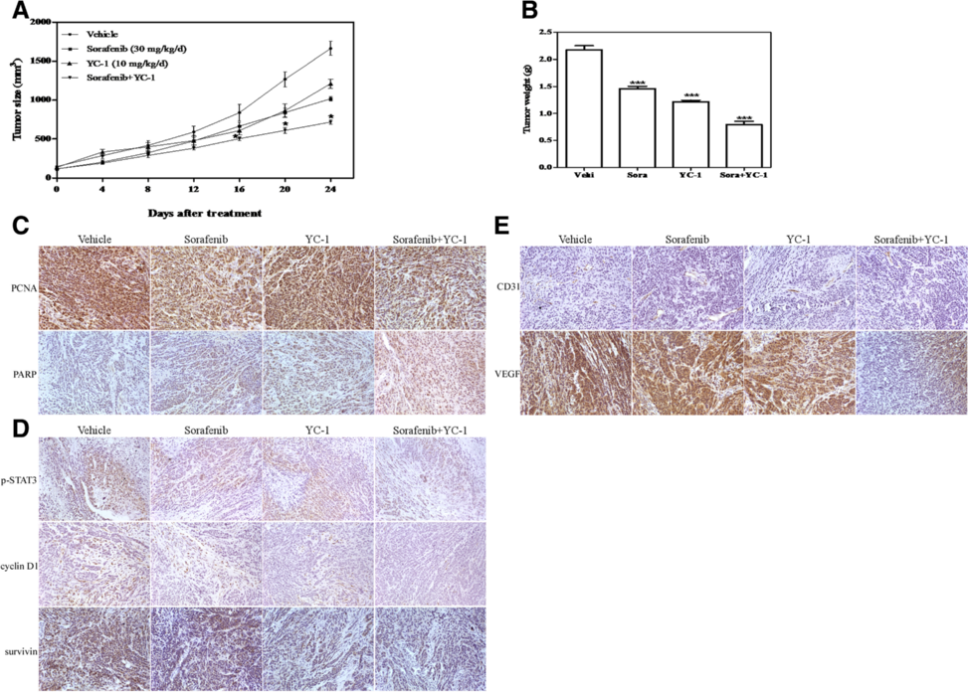
**Combination of sorafenib and YC-1 inhibited growth of HepG2 tumor xenografts.** HepG2 cells were injected subcutaneously into the upper right flank region of nude mice. When the tumor reached a mean size 100 mm^3^, mice were treated with combination of sorafenib (30 mg/kg/day) and YC-1 (10 mg/kg/day) or either drug alone every day for up to the 24th day. **A**, Tumor size was measured with a caliper rule every 3 days. Data were presented as the mean tumor volumes of mice in both treatment and vehicle groups on the days post-treatment. **B**, Average tumor weight was shown at the end of the experiments. **C** and **D**, Tumor sections were stained for PCNA, PAPR, p-STAT3, cyclin D1 and survivin. Representive images of the immunohistochemistry assay were shown (200×). **E**, Tumor sections were stained for CD31 and VEGF. Representive images of the immunohistochemistry assay were shown (200×). Error bars represent the SEM of tumor weight. P value <0.05 was considered statistically significant; ***p < 0.001, **p < 0.01, *p < 0.05.

To evaluate the role of sorafenib-YC-1 combination on tumor growth *in vivo*, we examined their effects in a HepG2 ectopic HCC model and HCCLM3 orthotopic model. Sorafenib or YC-1 inhibited the growth of the HepG2 xenografts, and their combination exhibited an enhanced effect (Figure 4A). Similar results were observed in HCCLM3 orthotopic model (Additional file 7: Figure S7). The excised tumors from vehicle mice, mice treated with either sorafenib or YC-1 alone and their combination weighed approximately 2000 mg, 1500 mg, 1200 mg and 800 mg respectively, which suggested significant effect when the mice were treated with the combination of drugs (Figure 4B). When tumors were treated with the combination of sorafenib and YC-1, significant decreases of cell proliferation and increases of apoptosis were observed by PCNA and PARP (Figure 4C and Additional file 8: Figure S8). There were no apparent changes in liver, heart, kidney, levels of serum glutamic-pyruvic transaminase (GOT) and glutamic oxalacetic transaminase (GST) and body weight in the mice (Additional file 9: Figure S9A, B and D). But the mice treated with sorafenib alone showed significant decreased spleen volume (0.0702 ± 0.003247 g) compared with control group (0.1050 ± 0.007266 g), YC-1 group (0.0992 ± 0.000860 g) or sorafenib + YC-1 group (0.08660 ± 0.003530 g) (Additional file 9: Figure S9C). These data implied that the sorafenib-YC-1 may be a potential therapeutic combination for treating HCC, and relatively nontoxic to the animals.

### Sorafenib and YC-1 combination inhibited the STAT3 signaling associated proteins and tumor angiogenesis *in vivo*

Furthermore, treatment with the sorafenib and YC-1 combination led to a significant reduction in p-STAT3, cyclin D1, and survivin compared with sorafenib or YC-1 used alone (Figure 4D and Additional file 8: Figure S8). Endothelial-specific antigen CD31 and VEGF levels were also significantly lower in sorafenib-YC-1 combination-treated tumor xenografts (Figure 4E and Additional file 8: Figure S8).

## Discussion

HCC typically arises on the basis of cirrhosis and responds poorly to conventional cytotoxic chemotherapy [31]. This has led to a search for novel approaches for molecular targeted therapy. The RAF/MEK/ERK cascade is one of the principal RAS-regulated pathways. Raf expression has been reported to be increased in human HCC, and sorafenib was synthesized to molecularly target RAF in this vital pathway and has been shown to have anti-tumor activity against renal cell cancer and HCC. However, recent researches have shown that STAT3 is a vital target of sorafenib and its effect is not correlated to the repression to Raf-1 kinase [22,23]. In addition, sorafenib causes multiple toxicities and a large percent of patients need to be treated with a reduced dose or stop taking the drug for this reason [32]. So we wanted to search for new agents that could be combined with sorafenib to reduce the dose dosage of sorafenib and enhance its HCC growth inhibition through enhanced suppressing STAT3 activity. In the present study, we showed not only the combined anti-tumor effects of low-dose sorafenib and YC-1 therapy against HCC both *in vitro* and *in vivo*, but also the novel mechanism through which YC-1 sensitized HCC cells to sorafenib. Our results indicated that the sorafenib-YC-1 combination possesses potential as a promising therapeutic agent against HCC.

Sorafenib is the first and only molecular targeted therapy approved for HCC and could inhibit proliferation of HCC cells by inducing apoptosis [33]. However, the poor response rate renders the drug less than satisfactory. In this study, YC-1 was used to enhance its inhibition. We found that sorafenib-YC-1 combination synergistically inhibited proliferation of HCC cells. Apoptosis and cell cycle analysis was further used to elucidate the mechanism involved in the synergistical effect of sorafenib and YC-1. Combination of sorafenib and YC-1 induced more apoptosis (23.99%) compared with sorafenib (16.77%) or YC-1 (5.1%) alone. Apoptosis rate was limited, which may not explain synergistical effect of sorafenib and YC-1. Our data demonstrated that YC-1 significantly induced S cell cycle arrest compared with the vehicle, and sorafenib increased the S cell cycle arrest of YC-1 in all HCC cells. Interestingly, Eun-Jin Yeo et al. (2006) suggested that YC-1 induced S cell cycle arrest and apoptosis by activating checkpoint kinases in Hep3B cells [18]. However, Wang SW et al. (2005) showed that YC-1 exhibited an anti-proliferative effect and arrested the cell cycle in G0-G1 in human HCC cells [19]. The reasons may be contributed to the different doses of YC-1 in the studies. Our results were consistent with Eun-jin Yeo el al. and further suggested that sorafenib could enhance the S cell cycle arrest of YC-1. Our data indicated that apoptosis and S cell cycle induced by sorafenib-YC-1 combination may be the mechanism of their synergistical inhibition of proliferation in human HCC cells.

**Figure 5:**
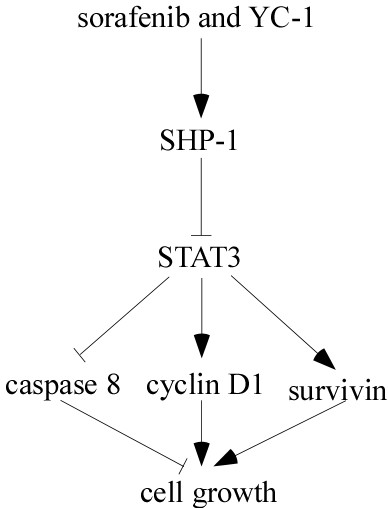
Association of between combination of sorafenib and YC-1 and STAT3 cascade.

STAT3 has a critical role in liver inflammation and tumor progression because it can be triggered by cytokines and growth factors such EGFR, FGFR and PDGF through tyrosine phosphorylation [34]. Many STAT3-related genes such as cyclin D1 and survivin also play roles in cell proliferation and survival signaling [35]. Constitutive activation of STAT3 is observed in 72.4% of human HCC and in a wide variety of other cancer types not in normal cells [36], which represents an attractive molecular target. In HCC, STAT3 frequently correlates with the constitutive upregulation of Ras implicated in HCC progression [37]. The anti-tumor activity of sorafenib does not necessarily correlate with the inhibition of ERK phosphorylation in HCC cells [25]. Previous studies also reported that STAT3 was the key factor in the mechanism of sorafenib resistance, and STAT3 rather than Raf-1 was critical in the anti-cancer effect of sorafenib [30]. Our studies showed that YC-1 or sorafenib alone suppressed the activity of STAT3 in a time- and dose-dependent manner. Furthermore, combination of sorafenib and YC-1 significantly inhibited the p-STAT3 (Y705) (S727) compared with sorafenib or YC-1 alone. Meantime, YC-1 did not inhibit the activity of p-ERK1/2 or only performed at a high dose (50 μM), which may be not significant in the clinic. However, combination of sorafenib and YC-1 significantly suppressed p-ERK1/2 in all tested HCC cell lines, whose mechanism needed to be determined in the future. Sorafenib suppresses the activity of STAT3 through enhancing SHP-1 activity [30]. Our results showed that combination of sorafenib and YC-1 may suppress the expression of p-STAT3 through inhibiting the of SHP-1 activity. And sorafenb-YC-1 combination also significantly suppressed the expression of cyclin D1 and survivin. These results indicated that YC-1 may be used to enhance the inhibition of STAT3 activity of sorafenib, and further suppress the HCC growth (Figure 5).

We examined the *in vivo* effect of sorafenib-YC-1 combination in HCC xenografts in nude mice. We demonstrated that combination of sorafenib (30 mg/kg/d) and YC-1 (10 mg/kg/d) could significantly suppress the growth of HCC tumor when compared with either drug alone. Of note, apparent toxicity was not observed in heart, lung, liver and kidney. But shrank spleen were found in the sorafenib group and the mechanism involved should be further elucidated to assure the safe use. Also, the treatment allowed the mice to maintain normal weight gain and levels of serum GOP and GPT. Our studies showed that combination of sorafenib and YC-1 could be safe to be used *in vivo*, which provided the preliminary basis for potential use for HCC patients in the future. Furthermore, combination of sorafenib and YC-1 also suppressed the expression of VEGF and microvessel density (CD31) in HCC tumor compared with either drug alone. VEGF is the most potent angiogenic factor and plays a key role in tumor associated angiogenesis and hyper-permeability [38]. Studies have shown that VEGF is frequently expressed in HCC [29]. Our data suggested that sorafenib-YC-1 combination to inhibit VEGF and CD31 expression may be another molecular mechanism to prevent HCC growth. Moreover, Liang et al. recently showed that anti-angiogenic activity of sorafenib could be responsible for the activation of resistance mechanisms, sustained sorafenib treatment led to increased intratuour hypoxia and induction of HIF-1α expression that mediated cell survival, and the use of HIF-1α inhibitors (EF24) abolished drug resistance [39]. YC-1 is also a HIF-1α inhibitor, so YC-1 may play a assisting role in anti-tumor of sorafenib through the mechanism above, which confirms us that sorafenib-YC-1 combination could be potential for use for HCC patients in near future.

## Conclusions

Our results revealed that YC-1 has a synergistic effect with sorafenib on HCC through inhibition of STAT3 activity and that the STAT3 signaling pathway may be a suitable target for the development of anti-HCC targeted agents.

## Competing interests

The authors declare that they have no competing interests.

## Authors' contributions

JK and FDK carried out the experiments and drafted the manuscript. JG, QBZ, FG, BP and SYD participated in the sequence alignment. LMZ and WBS conceived the study and coordination and helped to draft the manuscript. SK, QS and HCS participated in the design of the study. All authors read and approved the final manuscript.

## Supplementary Material

Additional 1: Figure S1 Chemical structures of sorafenib and YC-1 were shown. Click here for file

Additional 2: Figure S2 The effect of sorafenib and YC-1 on the proliferation of L02 cells. L02 cells were incubated with sorafenib (0–5 μmol/L) and/or YC-1 (0–20 μmol/L) for up to 72 h. Data were presented as percentages of cell proliferation as determined by CCK-8 assays. Click here for file

Additional 3: Figure S3 Combination of sorafenib and YC-1 induced S cell cycle arrest and apoptosis of HCC cells. BEL-7402 and HCCLM3 cells were treated with combination of sorafenib (5 μmol/L) and YC-1 (20 μmol/L) or either drug for 48 h. A, Cell cycle was assayed by PI/RNAse staining in BEL-7402 and HCCLM3 cells. B, The treated cells were assayed for apoptosis by annexin V/PI staining in BEL-7402 cells. C, Apoptosis in situ was assessed by Hoechst 33258 staining in BEL-7402 and HCCLM3 cells. Click here for file

Additional 4: Figure S4 Sorafenib and YC-1 affected STAT3 and ERK1/2 pathways. HepG2, BEL-7402 and HCCLM3 cells were treated with increasing doses of sorafenib (0–20 μmol/L) or YC-1 (0–50 μmol/L) for up to 4 h or 24 h. Cell extracts were subjected to western blot analysis and expression of p-ERK1/2, ERK2, p-STAT3 (Y705), STAT3 was detected. Actin served as loading control. Click here for file

Additional 5: Figure S5 Combination of sorafenib and YC-1 suppressed the activity of STAT3 (S727). HepG2, BEL-7402 and HCCLM3 cells were incubated with sorafenib (5 μmol/L) in combination with YC-1 (10 μmol/L or 20 μmol/L) or either drug alone for 24 h. Cell extracts were subjected to western blot analysis and expression of p-STAT3 (S727) and STAT3 was assessed. Actin served as loading control. Click here for file

Additional 6: Figure S6 Down-regulation of STAT3 sensitized HCC cells to combination of sorafenib and YC-1. A, siRNA was used to silence the expression of STAT3 in HepG2 and BEL-7402 cells. The expression of p-STAT3 (Y705) and STAT3 was assessed by western blot. Actin served as loading control. B, After HepG2 and BEL-7402 cells were transferred with STAT3 siRNA, sorafenib and YC-1 were added to treat the HepG2 and BEL-7402 cells. Apoptosis cells were analyzed by annexin V/PI staining. Click here for file

Additional 7: Figure S7 Combination of sorafenib and YC-1 inhibited growth of orthotopic HCCLM3 tumor. HCCLM3 cells were injected subcutaneously into the upper right flank region of nude mice. When the subcutaneous tumor reached approximately 1 cm in length, it was minced into small pieces of equal volume, and transplanted into the livers of 20 nude mice. When the tumor reached a mean size of about 100 mm^3^, mice were treated with combination of sorafenib (30 mg/kg/day) and YC-1 (10 mg/kg/day) or either drug alone every day for up to the 24th day. Representative image of tumor volume was displayed. Click here for file

Additional 8: Figure S8 The expression of PCNA, PARP, p-STAT3 (Y705), cyclin D1, survivin, CD31 and VEGF was quantified by Image-Pro Plus. Click here for file

Additional 9: Figure S9 Toxicity of sorafenib and/or YC-1 on nude mice bearing with tumor. HepG2 cells were injected subcutaneously into the upper right flank region of nude mice. When the tumor reached a mean size of about 100 mm^3^, mice were treated with combination of sorafenib (30 mg/kg/day) and YC-1 (10 mg/kg/day) or either drug alone every day for up to the 24th day. A, Mice weight was measured with a scale every 3 days. B, The levels of serum GOP and GPT were shown at the end of the experiments. Error bars represent the SEM of concentration of GOP and GPT. C, Spleen weight was measured at the end of the experiments. D, Heart, lung, liver and kidney sections were stained with haematoxylin and eosin (HE). Representive images were shown (200×). ns, no significance. Click here for file
