# Supplementary figures and images for: YC-1 enhances the anti-tumor activity of sorafenib through inhibition of signal transducer and activator of transcription 3 (STAT3) in hepatocellular carcinoma
Source: Mol Cancer. 2014 Jan 13;13:7. doi: 10.1186/1476-4598-13-7 (PMC3895679; doi:10.1186/1476-4598-13-7)

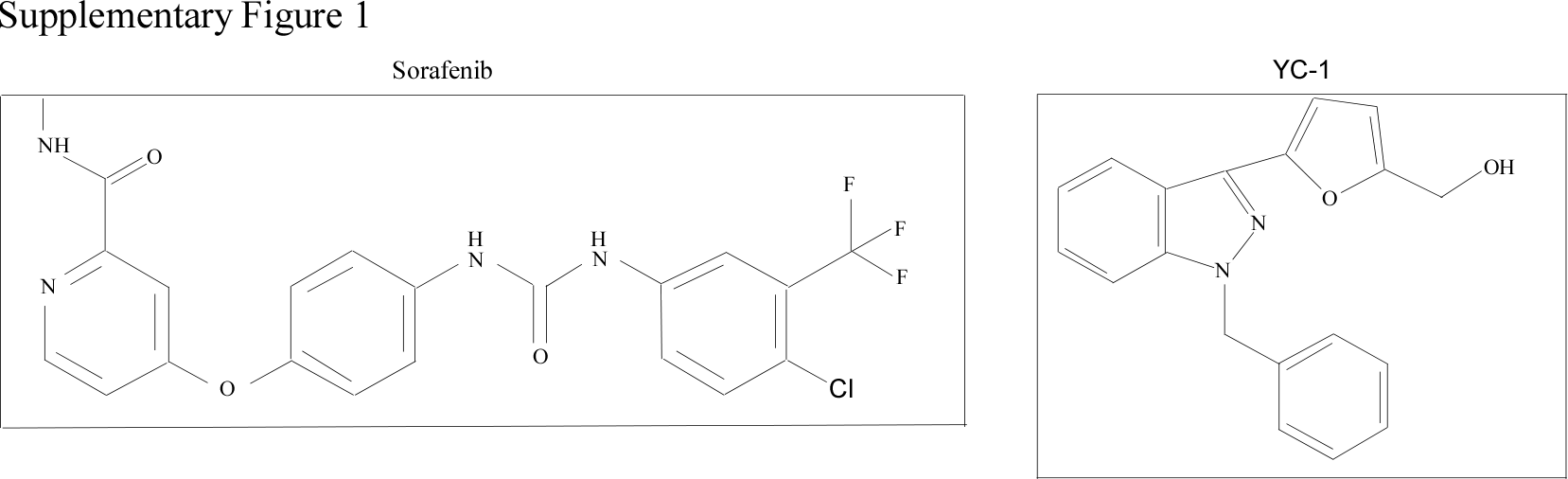


Supplementary Figure 1 - Chemical structures of sorafenib and YC-1 were shown.

Supplement: Additional 1: Figure S1 — Chemical structures of sorafenib and YC-1 were shown. [file 1476-4598-13-7-S1.doc]
