# Supplementary material for: YC-1 enhances the anti-tumor activity of sorafenib through inhibition of signal transducer and activator of transcription 3 (STAT3) in hepatocellular carcinoma
Source: Mol Cancer. 2014 Jan 13;13:7. doi: 10.1186/1476-4598-13-7 (PMC3895679; doi:10.1186/1476-4598-13-7)
Supplement: Additional 2: Figure S2 — The effect of sorafenib and YC-1 on the proliferation of L02 cells. L02 cells were incubated with sorafenib (0–5 μmol/L) and/or YC-1 (0–20 μmol/L) for up to 72 h. Data were presented as percentages of cell proliferation as determined by CCK-8 assays. [file 1476-4598-13-7-S2.doc]

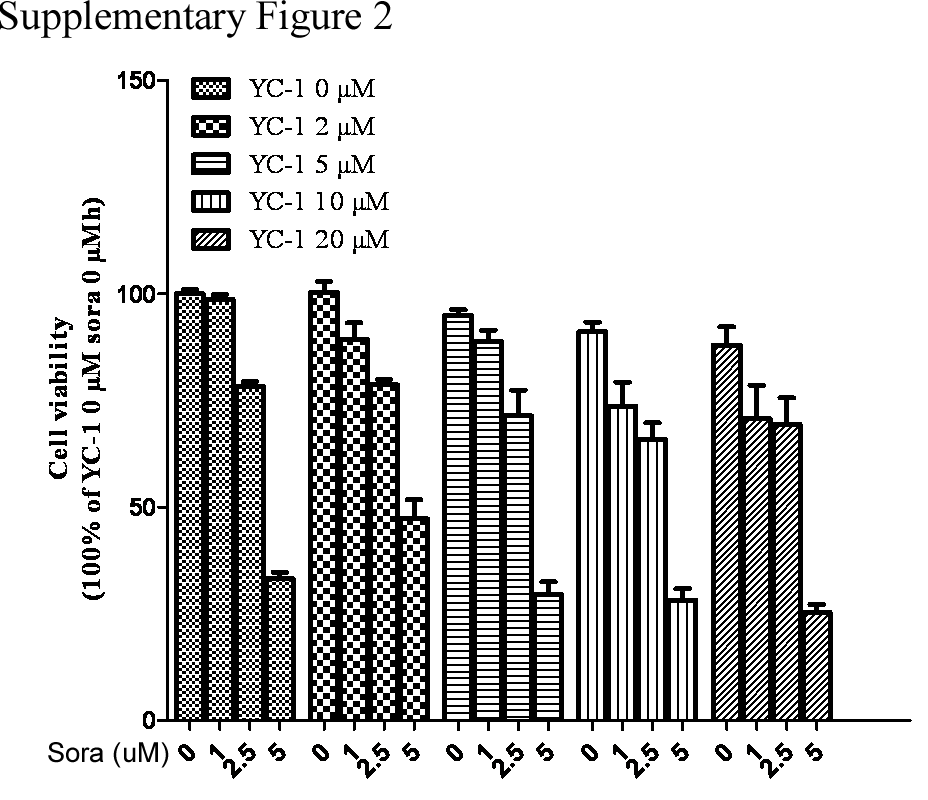


Supplementary Figure 2 – The effect of sorafenib and YC-1 on the proliferation of L02 cells. L02 cells were incubated with sorafenib (0-5 μmol/L) and/or YC-1 (0-20 μmol/L) for up to 72 h. Data were presented as percentages of cell proliferation as determined by CCK-8 assays.
