# Supplementary material for: YC-1 enhances the anti-tumor activity of sorafenib through inhibition of signal transducer and activator of transcription 3 (STAT3) in hepatocellular carcinoma
Source: Mol Cancer. 2014 Jan 13;13:7. doi: 10.1186/1476-4598-13-7 (PMC3895679; doi:10.1186/1476-4598-13-7)
Supplement: Additional 3: Figure S3 — Combination of sorafenib and YC-1 induced S cell cycle arrest and apoptosis of HCC cells. BEL-7402 and HCCLM3 cells were treated with combination of sorafenib (5 μmol/L) and YC-1 (20 μmol/L) or either drug for 48 h. A, Cell cycle was assayed by PI/RNAse staining in BEL-7402 and HCCLM3 cells. B, The treated cells were assayed for apoptosis by annexin V/PI staining in BEL-7402 cells. C, Apoptosis in situ was assessed by Hoechst 33258 staining in BEL-7402 and HCCLM3 cells. [file 1476-4598-13-7-S3.doc]

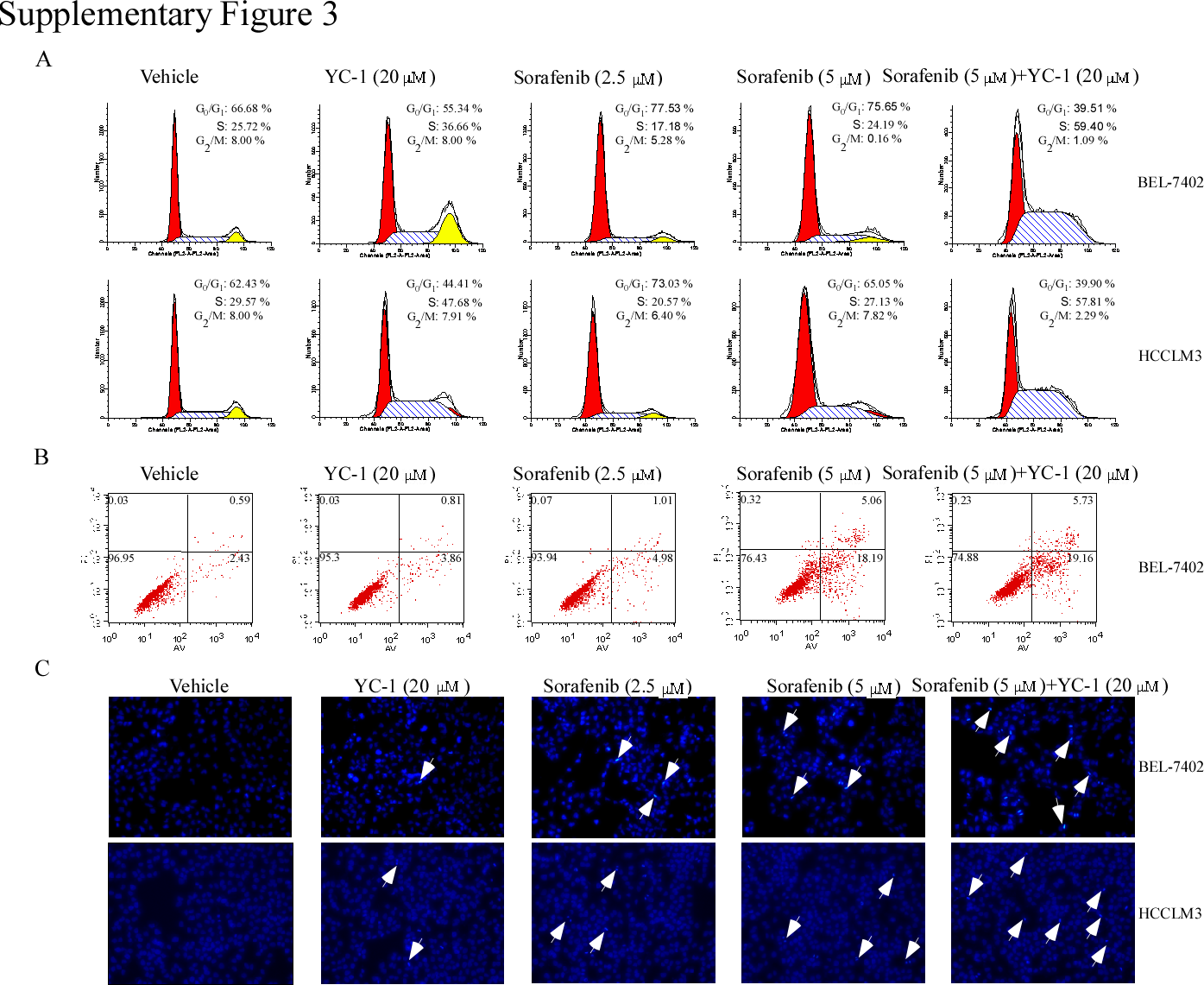


Supplementary Figure 3 - Combination of sorafenib and YC-1 induced S cell cycle arrest and apoptosis of HCC cells. BEL-7402 and HCCLM3 cells were treated with combination of sorafenib (5 μmol/L) and YC-1 (20 μmol/L) or either drug for 48 h. A, Cell cycle was assayed by PI/RNAse staining in BEL-7402 and HCCLM3 cells. B, The treated cells were assayed for apoptosis by annexin Ⅴ/PI staining in BEL-7402 cells. C, Apoptosis in situ was assessed by Hoechst 33258 staining in BEL-7402 and HCCLM3 cells.
