# Supplementary material for: YC-1 enhances the anti-tumor activity of sorafenib through inhibition of signal transducer and activator of transcription 3 (STAT3) in hepatocellular carcinoma
Source: Mol Cancer. 2014 Jan 13;13:7. doi: 10.1186/1476-4598-13-7 (PMC3895679; doi:10.1186/1476-4598-13-7)
Supplement: Additional 4: Figure S4 — Sorafenib and YC-1 affected STAT3 and ERK1/2 pathways. HepG2, BEL-7402 and HCCLM3 cells were treated with increasing doses of sorafenib (0–20 μmol/L) or YC-1 (0–50 μmol/L) for up to 4 h or 24 h. Cell extracts were subjected to western blot analysis and expression of p-ERK1/2, ERK2, p-STAT3 (Y705), STAT3 was detected. Actin served as loading control. [file 1476-4598-13-7-S4.doc]

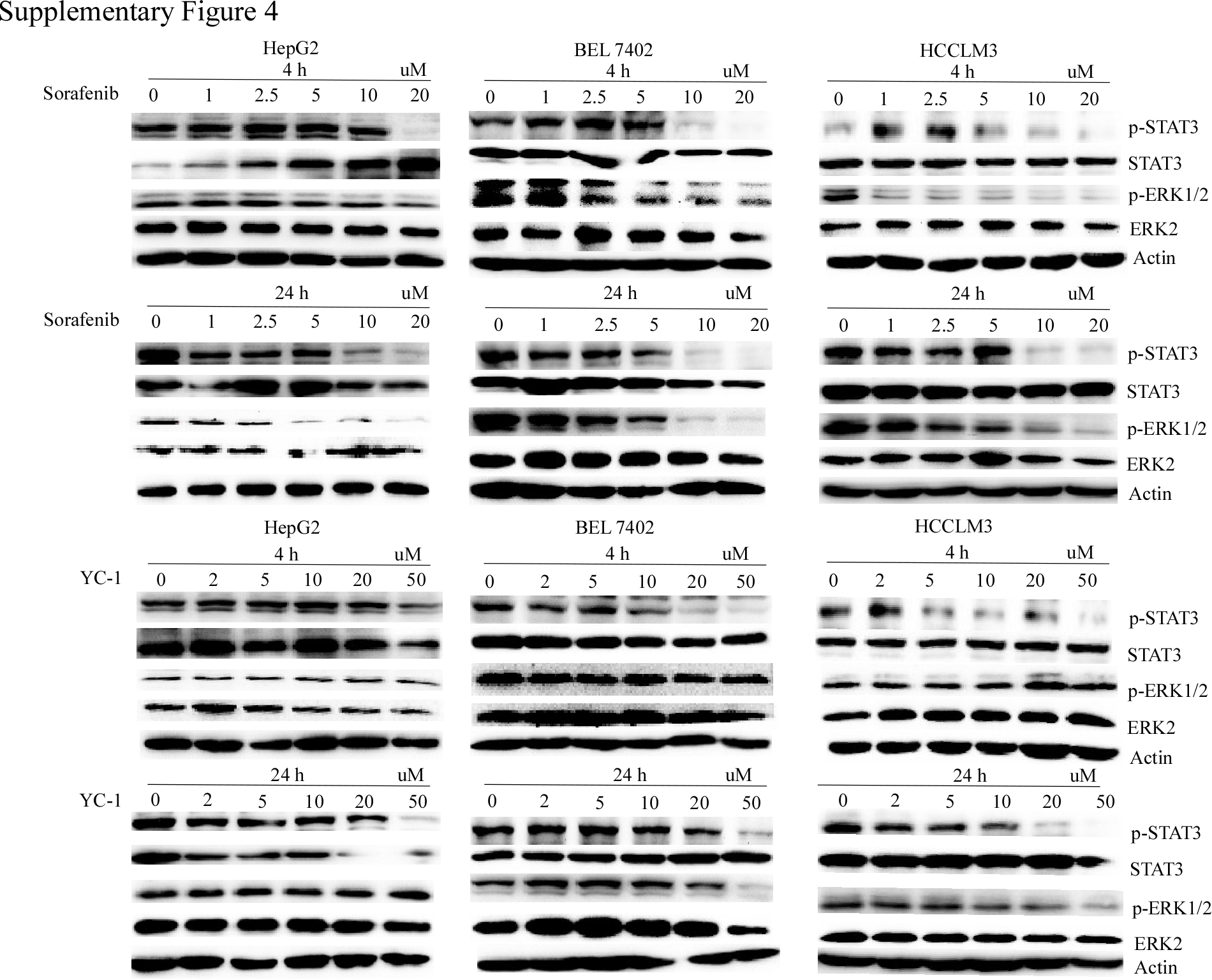


Supplementary Figure 4 -Sorafenib and YC-1 affected STAT3 and ERK1/2 pathways. HepG2, BEL-7402 and HCCLM3 cells were treated with increasing doses of sorafenib (0-20 μmol/L) or YC-1 (0-50 μmol/L) for up to 4 h or 24 h. Cell extracts were subjected to western blot analysis and expression of p-ERK1/2, ERK2, p-STAT3 (Y705), STAT3 was detected. Actin served as loading control.
