# Supplementary material for: YC-1 enhances the anti-tumor activity of sorafenib through inhibition of signal transducer and activator of transcription 3 (STAT3) in hepatocellular carcinoma
Source: Mol Cancer. 2014 Jan 13;13:7. doi: 10.1186/1476-4598-13-7 (PMC3895679; doi:10.1186/1476-4598-13-7)
Supplement: Additional 5: Figure S5 — Combination of sorafenib and YC-1 suppressed the activity of STAT3 (S727). HepG2, BEL-7402 and HCCLM3 cells were incubated with sorafenib (5 μmol/L) in combination with YC-1 (10 μmol/L or 20 μmol/L) or either drug alone for 24 h. Cell extracts were subjected to western blot analysis and expression of p-STAT3 (S727) and STAT3 was assessed. Actin served as loading control. [file 1476-4598-13-7-S5.doc]

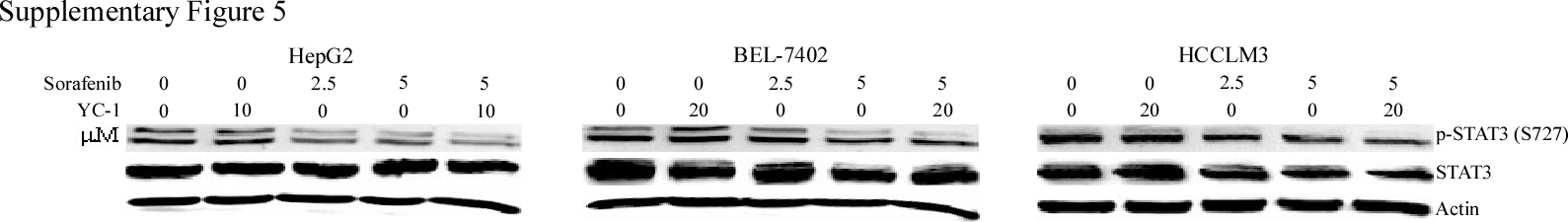


Supplementary Figure 5 - Combination of sorafenib and YC-1 suppressed the activity of STAT3 (S727). HepG2, BEL-7402 and HCCLM3 cells were incubated with sorafenib (5 μmol/L) in combination with YC-1 (10 μmol/L or 20 μmol/L) or either drug alone for 24 h. Cell extracts were subjected to western blot analysis and expression of p-STAT3 (S727) and STAT3 was assessed. Actin served as loading control.
