# Supplementary material for: YC-1 enhances the anti-tumor activity of sorafenib through inhibition of signal transducer and activator of transcription 3 (STAT3) in hepatocellular carcinoma
Source: Mol Cancer. 2014 Jan 13;13:7. doi: 10.1186/1476-4598-13-7 (PMC3895679; doi:10.1186/1476-4598-13-7)
Supplement: Additional 6: Figure S6 — Down-regulation of STAT3 sensitized HCC cells to combination of sorafenib and YC-1. A, siRNA was used to silence the expression of STAT3 in HepG2 and BEL-7402 cells. The expression of p-STAT3 (Y705) and STAT3 was assessed by western blot. Actin served as loading control. B, After HepG2 and BEL-7402 cells were transferred with STAT3 siRNA, sorafenib and YC-1 were added to treat the HepG2 and BEL-7402 cells. Apoptosis cells were analyzed by annexin V/PI staining. [file 1476-4598-13-7-S6.doc]

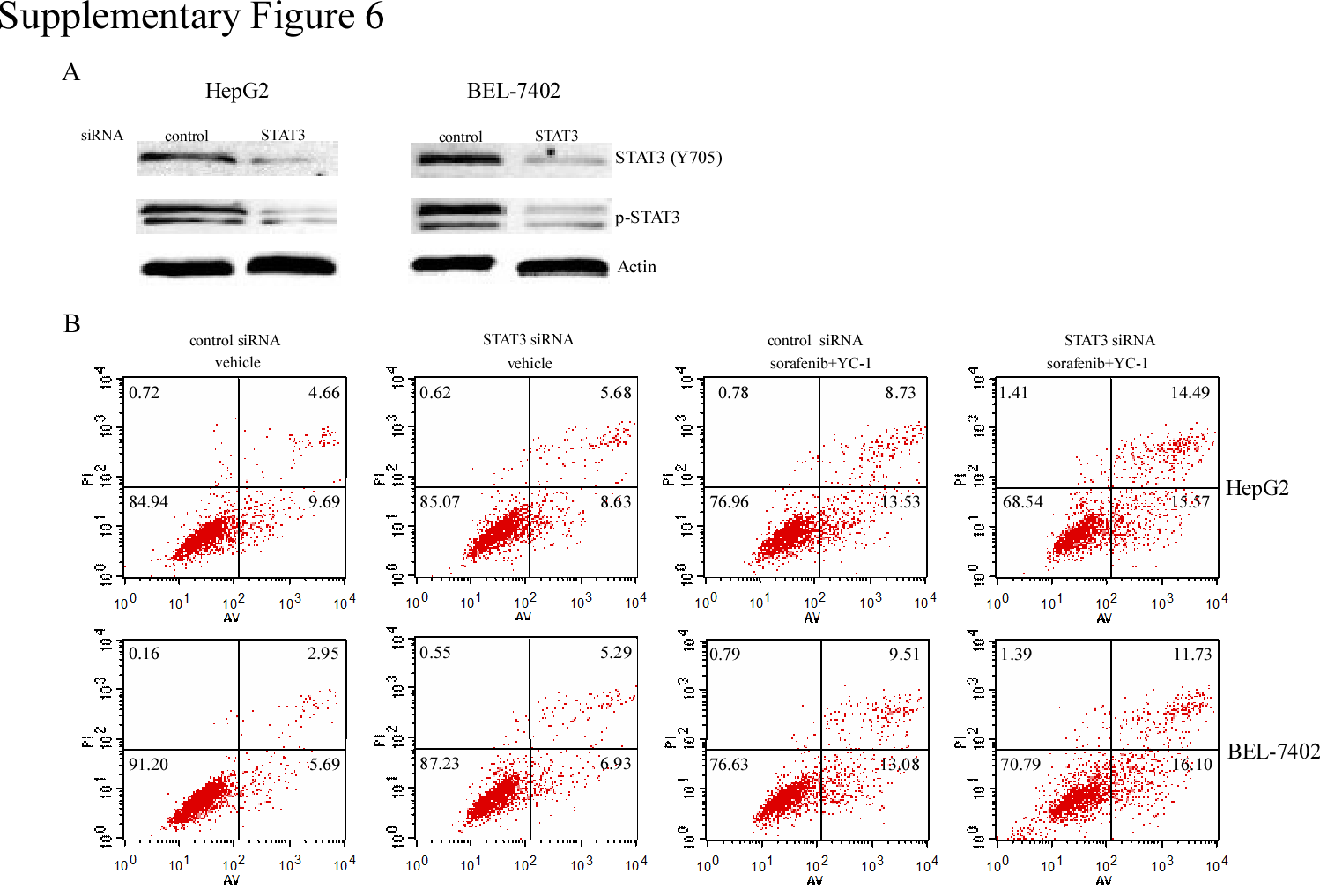


Supplementary Figure 6 – Down-regulation of STAT3 sensitized HCC cells to combination of sorafenib and YC-1. A, siRNA was used to silence the expression of STAT3 in HepG2 and BEL-7402 cells. The expression of p-STAT3 (Y705) and STAT3 was assessed by western blot. Actin served as loading control. B, After HepG2 and BEL-7402 cells were transferred with STAT3 siRNA, sorafenib and YC-1 were added to treat the HepG2 and BEL-7402 cells. Apoptosis cells were analyzed by annexin Ⅴ/PI staining.
