# Supplementary material for: YC-1 enhances the anti-tumor activity of sorafenib through inhibition of signal transducer and activator of transcription 3 (STAT3) in hepatocellular carcinoma
Source: Mol Cancer. 2014 Jan 13;13:7. doi: 10.1186/1476-4598-13-7 (PMC3895679; doi:10.1186/1476-4598-13-7)
Supplement: Additional 7: Figure S7 — Combination of sorafenib and YC-1 inhibited growth of orthotopic HCCLM3 tumor. HCCLM3 cells were injected subcutaneously into the upper right flank region of nude mice. When the subcutaneous tumor reached approximately 1 cm in length, it was minced into small pieces of equal volume, and transplanted into the livers of 20 nude mice. When the tumor reached a mean size of about 100 mm3, mice were treated with combination of sorafenib (30 mg/kg/day) and YC-1 (10 mg/kg/day) or either drug alone every day for up to the 24th day. Representative image of tumor volume was displayed. [file 1476-4598-13-7-S7.doc]

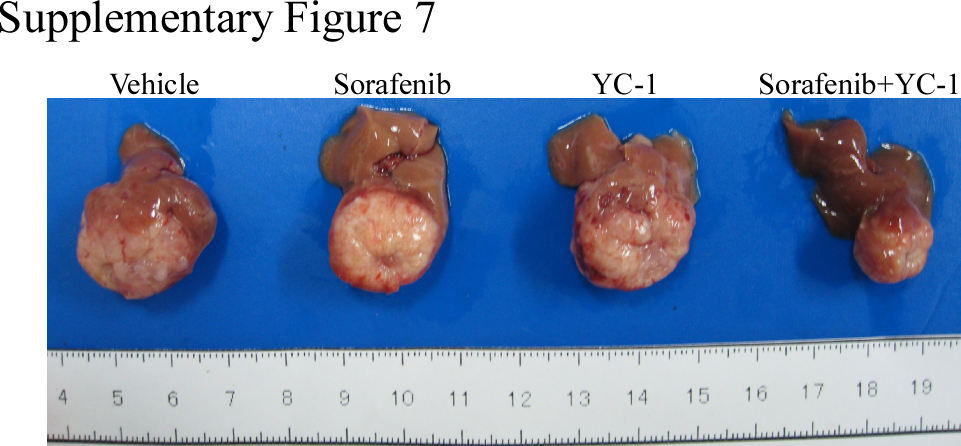


Supplementary Figure 7 - Combination of sorafenib and YC-1 inhibited growth of orthotopic HCCLM3 tumor. HCCLM3 cells were injected subcutaneously into the upper right flank region of nude mice. When the subcutaneous tumor reached approximately 1 cm in length, it was minced into small pieces of equal volume, and transplanted into the livers of 20 nude mice. When the tumor reached a mean size of about 100 mm^3^, mice were treated with combination of sorafenib (30 mg/kg/day) and YC-1 (10 mg/kg/day) or either drug alone every day for up to the 24^th^ day. Representative image of tumor volume was displayed.
