# Supplementary material for: YC-1 enhances the anti-tumor activity of sorafenib through inhibition of signal transducer and activator of transcription 3 (STAT3) in hepatocellular carcinoma
Source: Mol Cancer. 2014 Jan 13;13:7. doi: 10.1186/1476-4598-13-7 (PMC3895679; doi:10.1186/1476-4598-13-7)
Supplement: Additional 8: Figure S8 — The expression of PCNA, PARP, p-STAT3 (Y705), cyclin D1, survivin, CD31 and VEGF was quantified by Image-Pro Plus. [file 1476-4598-13-7-S8.doc]

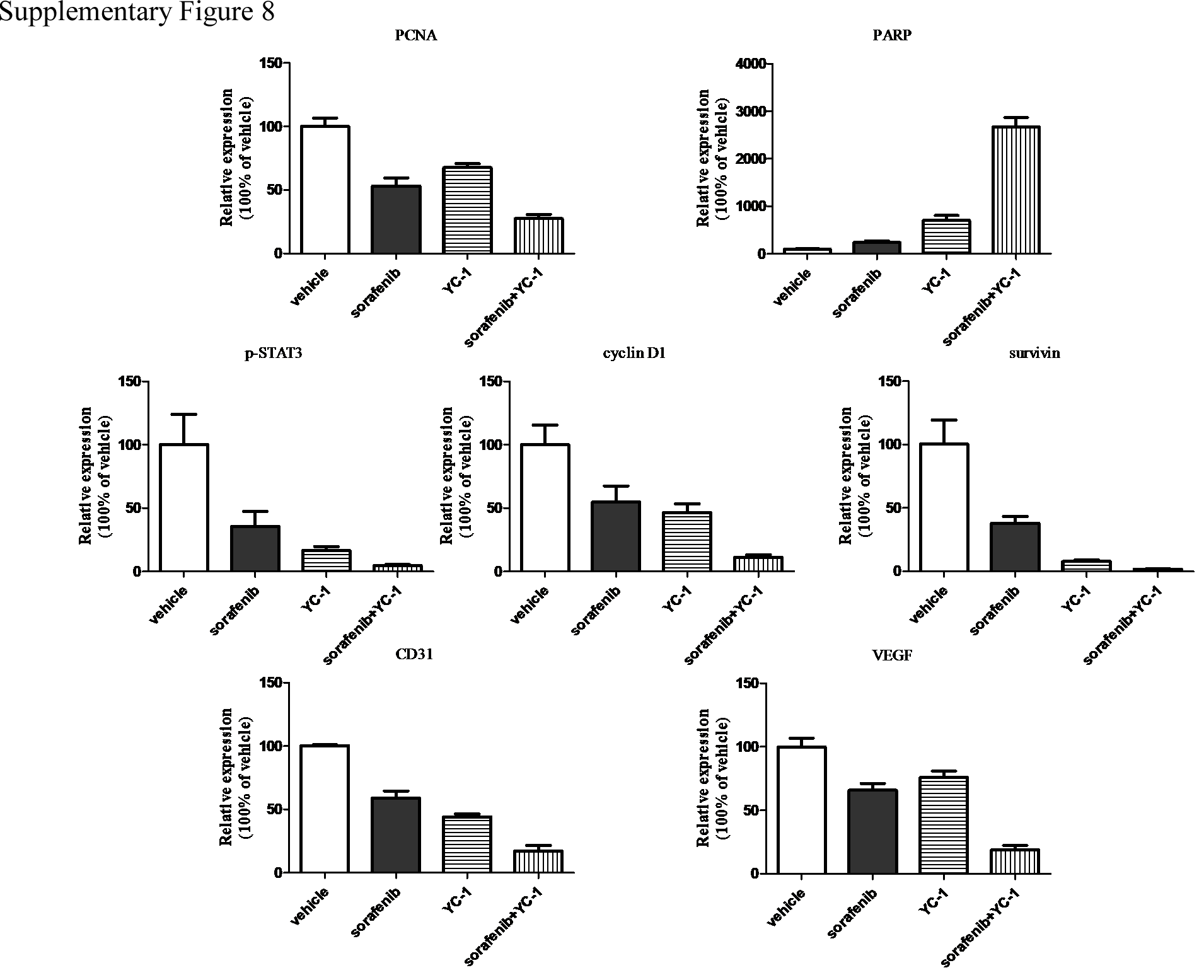


Supplementary Figure 8 – The expression of PCNA, PARP, p-STAT3 (Y705), cyclin D1, survivin, CD31 and VEGF was quantified by Image-Pro Plus.
