# Supplementary material for: YC-1 enhances the anti-tumor activity of sorafenib through inhibition of signal transducer and activator of transcription 3 (STAT3) in hepatocellular carcinoma
Source: Mol Cancer. 2014 Jan 13;13:7. doi: 10.1186/1476-4598-13-7 (PMC3895679; doi:10.1186/1476-4598-13-7)
Supplement: Additional 9: Figure S9 — Toxicity of sorafenib and/or YC-1 on nude mice bearing with tumor. HepG2 cells were injected subcutaneously into the upper right flank region of nude mice. When the tumor reached a mean size of about 100 mm3, mice were treated with combination of sorafenib (30 mg/kg/day) and YC-1 (10 mg/kg/day) or either drug alone every day for up to the 24th day. A, Mice weight was measured with a scale every 3 days. B, The levels of serum GOP and GPT were shown at the end of the experiments. Error bars represent the SEM of concentration of GOP and GPT. C, Spleen weight was measured at the end of the experiments. D, Heart, lung, liver and kidney sections were stained with haematoxylin and eosin (HE). Representive images were shown (200×). ns, no significance. [file 1476-4598-13-7-S9.doc]

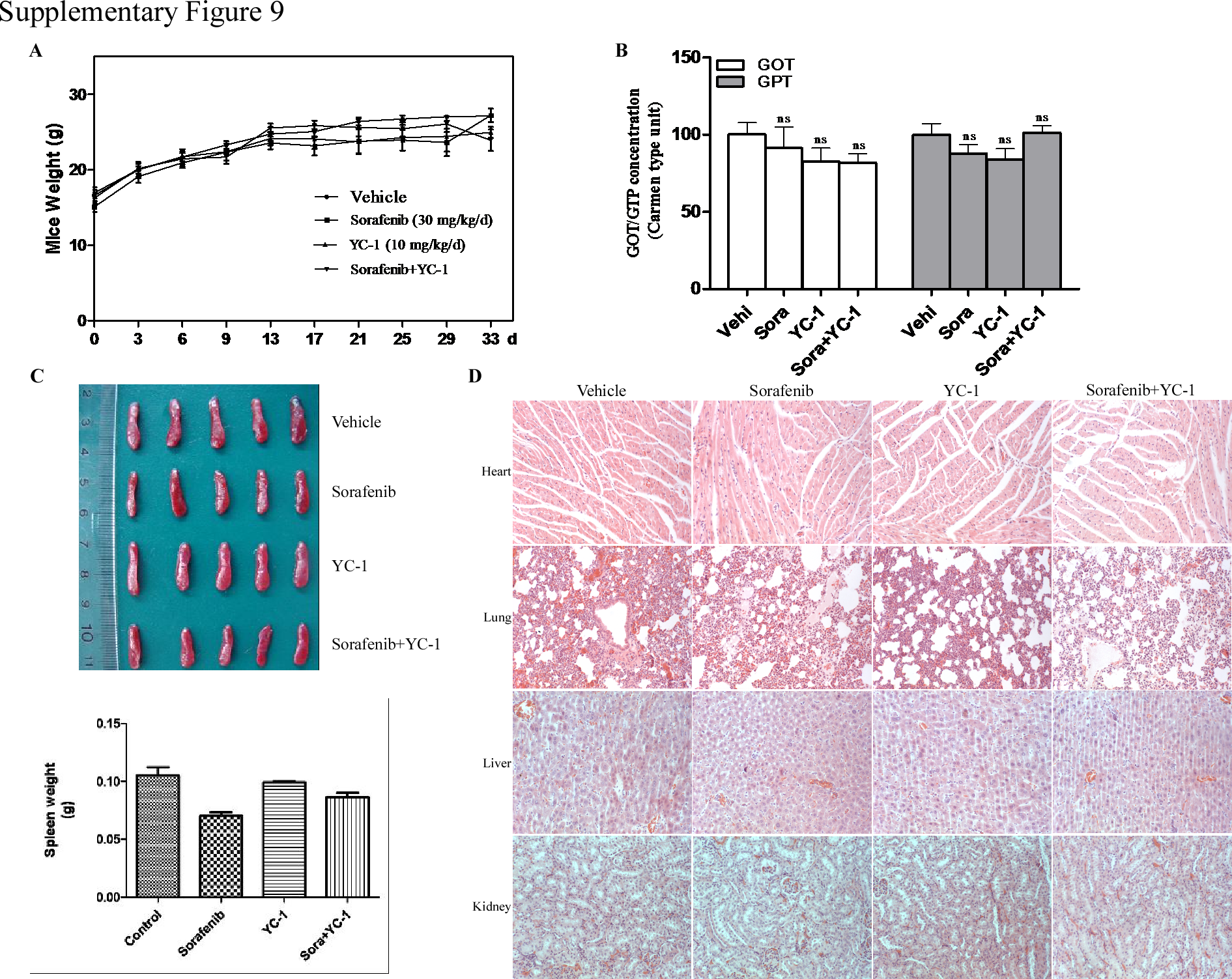


Supplementary Figure 9 - Toxicity of sorafenib and/or YC-1 on nude mice bearing with tumor. HepG2 cells were injected subcutaneously into the upper right flank region of nude mice. When the tumor reached a mean size of about 100 mm^3^, mice were treated with combination of sorafenib (30 mg/kg/day) and YC-1 (10 mg/kg/day) or either drug alone every day for up to the 24^th^ day. A, Mice weight was measured with a scale every 3 days. B, The levels of serum GOP and GPT were shown at the end of the experiments. Error bars represent the SEM of concentration of GOP and GPT. C, Spleen weight was measured at the end of the experiments. D, Heart, lung, liver and kidney sections were stained with haematoxylin and eosin (HE). Representive images were shown (200×). ns, no significance.
